# Neuroprotective Effect of Cyclo-(L-Pro-L-Phe) Isolated from the Jellyfish-Derived Fungus *Aspergillus flavus*

**DOI:** 10.3390/md19080417

**Published:** 2021-07-26

**Authors:** Dan-dan Li, Ying Wang, Eun La Kim, Jongki Hong, Jee H. Jung

**Affiliations:** 1 College of Pharmacy, Pusan National University, Busan 46241, Korea; leedany1992@gmail.com (D.-d.L.); wangrunhe829@gmail.com (Y.W.); eunlakim@gmail.com (E.L.K.); 2 College of Pharmacy, Kyung Hee University, Seoul 02447, Korea; jhong@khu.ac.kr

**Keywords:** PPAR, 2,5-diketopiperazines, cyclo-(L-Pro-L-Phe), neuroprotection, oxidative stress

## Abstract

Peroxisome proliferator-activated receptor (PPAR) expression has been implicated in pathological states such as cancer, inflammation, diabetes, and neurodegeneration. We isolated natural PPAR agonists—eight 2,5-diketopiperazines—from the jellyfish-derived fungus *Aspergillus flavus*. Cyclo-(L-Pro-L-Phe) was the most potent PPAR-γ activator among the eight 2,5-DKPs identified. Cyclo-(L-Pro-L-Phe) activated PPAR-γ in Ac2F rat liver cells and SH-SY5Y human neuroblastoma cells. The neuroprotective effect of this partial PPAR-γ agonist was examined using the 3-(4, 5-dimethylthiazol-2-yl)-2,5-diphenyltetrazolium bromide assay, lactate dehydrogenase release, and the Hoechst 33342 staining assay in SH-SY5Y cells. Our findings revealed that cyclo-(L-Pro-L-Phe) reduced hydrogen peroxide-induced apoptosis as well as the generation of reactive oxygen species. Rhodamine 123 staining and western blotting revealed that cyclo-(L-Pro-L-Phe) prevented the loss of mitochondrial membrane potential and inhibited the activation of mitochondria-related apoptotic proteins, such as caspase 3 and poly (ADP-ribose) polymerase. Moreover, cyclo-(L-Pro-L-Phe) inhibited the activation and translocation of nuclear factor-kappa B. Thus, the partial PPAR-γ agonist cyclo-(L-Pro-L-Phe) demonstrated potential neuroprotective activity against oxidative stress-induced neurodegeneration in SH-SY5Y cells.

## 1. Introduction

Neurodegenerative diseases are a group of heterogeneous disorders characterized by a gradual loss of neuronal structure or function and include conditions such as Alzheimer’s disease (AD), Parkinson’s disease (PD), amyotrophic lateral sclerosis, and Huntington’s disease (HD) [1,2]. Accumulating evidence has indicated the potential role of oxidative stress, mitochondrial dysfunction, inflammation, autophagy, and apoptotic dysfunction in neurodegenerative diseases [3,4,5,6]. Notably, oxidative stress, which induces mitochondrial DNA damage, has been implicated as the primary underlying cause of neurodegenerative diseases, such as AD and PD [7].

Peroxisome proliferator-activated receptors (PPARs) are nuclear receptors that regulate ligand-activated transcription [8] and are known to include three isotypes: PPAR-α, β/δ, and γ [8,9]. PPAR-α is expressed in the brown adipose tissue, liver, kidney, heart, and brain [9,10]. In the brain, PPAR-α upregulates the expression of the gene coding α-secretase, which is known to mediate amyloid precursor protein degradation while it downregulates the expression of the gene coding β-secretase that mainly enhances amyloid-beta (Aβ) peptide release [10]. The inhibition of PPAR-α expression may alter mitochondrial function and suppress antioxidant and anti-inflammatory activities [10]. By contrast, the activation of PPAR-α expression via PPAR-α agonists, gemfibrozil, and WY14643 reportedly ameliorates spatial learning defects and memory impairment in AD mice [11]. PPAR-β/δ is expressed in the gut, kidney, heart, and brain [9,10]. Thus, PPAR-β/δ could mediate anti-oxidative and anti-inflammatory processes in the damaged brain and PPAR-β/δ deletion was found to induce developmental defects in the mouse brain [12]. In addition, PPAR-β/δ activation by the selective PPAR-β/δ agonist GW0742 exhibits a neuroprotective effect by suppressing inflammation and apoptosis in a mouse model [13]. PPAR-γ is expressed in the adipose tissue, liver, colon, and brain [9,10,14]. Reportedly, PPAR-γ activation stimulates neuronal differentiation and axon polarity [15]. PPAR-γ agonists can decrease the incidence of several neurological disorders [16] and impart protective effects against apoptosis, mitochondrial dysfunction, and oxidative damage [17]. The PPAR-γ agonist pioglitazone was found to improve learning and memory impairment in mice, as well as ameliorate cognitive impairment in diabetic patients with AD [18,19]. In human neural stem cells, rosiglitazone protects cells from Aβ-induced mitochondrial dysfunction and oxidative stress [20]. In a mouse model of traumatic brain injury, rosiglitazone exhibited neuroprotective effects by mediating anti-inflammatory, anti-apoptotic, and anti-oxidative activities [21]. Although several PPAR agonists exhibit neuroprotective effects in neurodegenerative diseases, such as PD, their applications are restricted given the high-dose requirement or toxic side effects [22]. Therefore, new PPAR agonists with fewer side effects need to be developed as potential therapeutic options in neurodegenerative diseases.

Nuclear factor-kappa B (NF-κB) is a transcription factor involved in inflammatory response and apoptosis [23,24]. NF-κB is widely expressed in the central nervous system (CNS) and is associated with IκB in its inactive form [25]. However, in CNS diseases, the NF-κB inhibitor IκB is phosphorylated and degraded following stimulation by several inducers that is followed by nuclear translocation and binding to inflammatory gene response elements [25]. In CNS diseases, PPAR agonists exert benefits by inhibiting the NF-κB pathway (inhibiting the activation of NF-κB or DNA binding of the activated NF-κB) [25]. Reportedly, the PPAR-γ agonist pioglitazone decreases NF-κB activation in a 6-hydroxydopamine induced PD model [26].

2,5-Diketopiperazines (2,5-DKPs), also known as cyclic dipeptides [27], are found to occur in diverse natural products [28] and demonstrate attractive bioactivities, including antitumor, neuroprotective, anti-inflammatory, and antibiotic activities [29,30,31,32,33,34,35,36]. Moreover, these compounds were found to possess high stability against proteolysis as well as superior intestinal absorption conferred by their unique heterocyclic structure [33]. Moreover, the donor and acceptor groups of the molecule can interact with biological targets through hydrogen bonding [37]. In the present study, we isolated eight 2,5-DKPs using bioactivity-guided fractionation of the jellyfish-derived fungus *Aspergillus flavus* (Figure S1). The luciferase assay revealed that cyclo-(L-Pro-L-Phe) was the most potent PPAR-γ activator among the eight 2,5-DKPs identified. Cyclo-(L-Pro-L-Phe) reportedly demonstrates antibacterial [38,39] and antifungal activities [40], along with cytotoxicity [41,42], and regulates plant growth activity [38,43,44]. To the best of our knowledge, no previous reports have revealed the biological activity of cyclo-(L-Pro-L-Phe) against oxidative stress (H_2_O_2_-induced neurodegeneration). Herein, we reported the neuroprotective effects of the partial PPAR-γ agonist cyclo-(L-Pro-L-Phe) against H_2_O_2_-induced damage in neuroblastoma cells.

## 2. Results and Discussion

### 2.1. Identification of 2,5-DKPs

**Figure 1:**
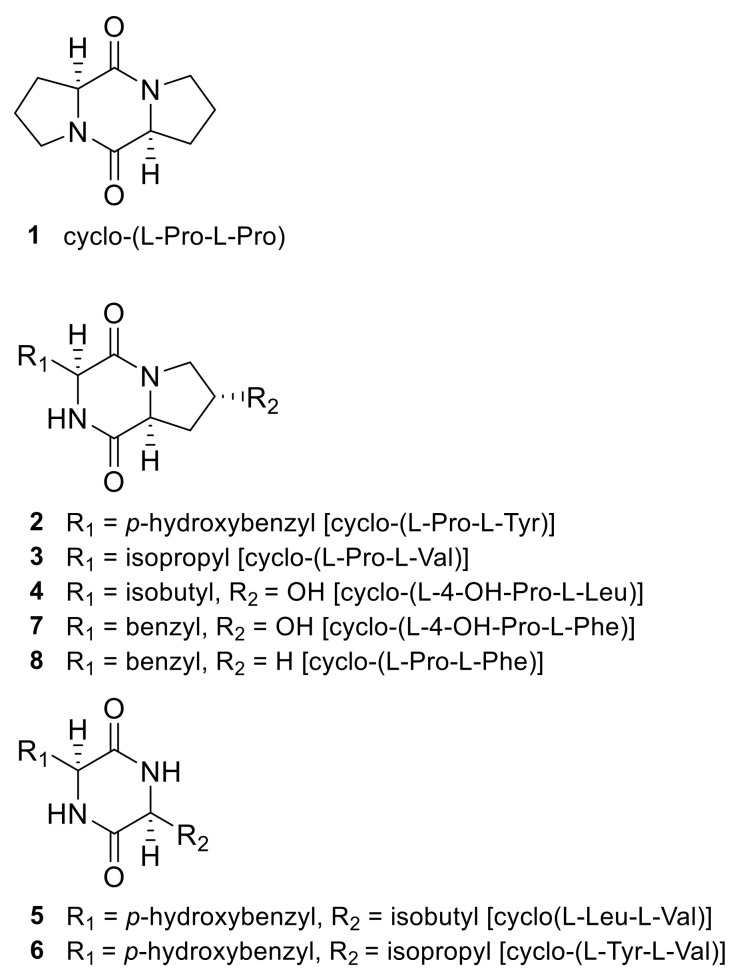
Structures of 2,5-diketopiperazines isolated from the jellyfish-derived fungus *Aspergillus flavus*.

Compounds **1–8** (Figure 1) were identified as cyclo-(L-Pro-L-Pro) (**1**) [45]; cyclo-(L-Pro-L-Tyr) (**2**) [46]; cyclo-(L-Pro-L-Val) (**3**) [45]; cyclo-(L-4-OH-Pro-L-Leu) (**4**) [45]; cyclo-(L-Leu-L-Tyr) (**5**) [47]; cyclo-(L-Tyr-L-Val) (**6**) [48]; cyclo-(L-4-OH-Pro-L-Phe) (**7**) [45]; and cyclo-(L-Pro-L-Phe) (**8**) [45,49] by comparing the nuclear magnetic resonance spectroscopy (^1^H-and ^13^C-NMR) results and optical rotation data with those in references.

### 2.2. PPAR Transactivation by 2,5-DKPs

**Figure 2:**
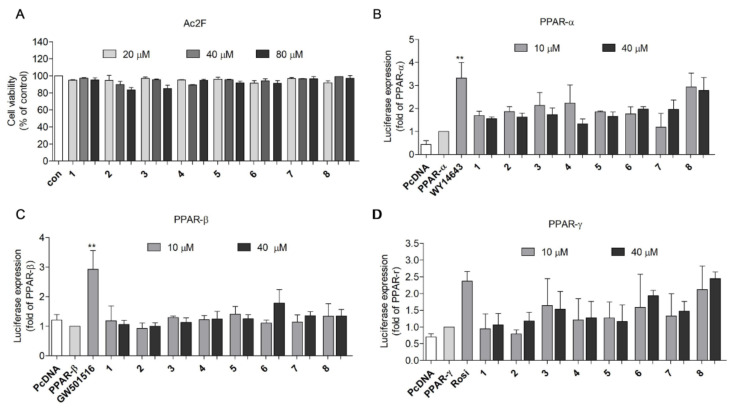
Transactivation effects of the derived compounds on PPAR-α, -β/δ, and -γ in Ac2F cells. (**A**) Cytotoxicity of derived compounds against Ac2F cells. Ac2F cells were treated with 2,5-diketopiperazines for 12 h in free medium and the cell viability was measured using the MTT assay. (**B**–**D**) Transactivation effects of compounds on PPAR-α, -β/δ, and –γ. Plasmids were transfected into the Ac2F cells and the effects of 2,5-diketopiperazine activation were measured using the luciferase assay. WY-14643, GW501516, and rosiglitazone were employed as the standard agonists of PPAR-α, -β/δ, and –γ, respectively. The cells transfected with PcDNA plasmid were used as blank. The cells transfected with PPRE together with PPAR-α; -β/δ or –γ plasmids were employed as controls. ** *p* < 0.01 vs. PPAR-α, or -β/δ. Rosi, rosiglitazone.

Prior to evaluating the PPAR agonistic activity of 2,5-DKPs, the cytotoxicity of 2,5-DKPs (20, 40, and 80 μM) was assessed using the 3-(4, 5-dimethylthiazol-2-yl)-2,5-diphenyltetrazolium bromide (MTT) assay to determine the appropriate concentration for the PPAR transactivation assay. No significant cytotoxicity was observed for all compounds in Ac2F rat liver cells up to 80 μM (Figure 2A). Concentrations of 10 and 40 μM were selected for PPAR (PPAR-α, PPAR-β/δ, and PPAR-γ) transactivation assays; WY-14643, GW501516, and rosiglitazone were used as positive controls for PPAR-α, PPAR-β/δ, and PPAR-γ activation, respectively. Cyclo-(L-Pro-L-Phe) (**8**) was the most potent PPAR-γ activator among the eight 2,5-DKPs identified. When compound **8** was compared to the PPAR-γ full agonist rosiglitazone, using one-way analysis of variance and Tukey’s HSD-post hoc test, the significance of activity was observed at 40 μM. Compound **8** exhibited mild PPAR-γ transactivation effects, with negligible effects on PPAR-β/δ (Figure 2B–D). This finding indicated that **8** could be a possible partial PPAR-γ agonist. Therefore, **8** was selected for further study.

**Figure 3:**
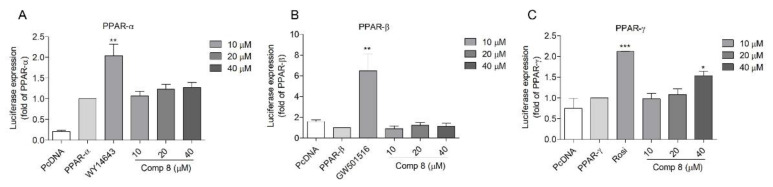
Transactivation effects of compound **8** on PPAR-α (**A**), -β/δ (**B**), and -γ (**C**) in SH-SY5Y cells. SH-SY5Y cells were transfected with PPAR-α, -β/δ, and -γ plasmid for 24 h and treated with **8** for another 24 h. The activation effects were measured using the luciferase assay. WY-14643, GW501516, and rosiglitazone were employed as the positive control for PPAR-α, -β/δ, and -γ, respectively. The cells transfected with PcDNA plasmid were used as a blank. The cells transfected with PPRE together with PPAR-α; -β/δ or –γ plasmids were employed as controls. * *p* < 0.05, ** *p* < 0.01, *** *p* < 0.001 vs. PPAR-α, -β/δ, or and -γ. Rosi, rosiglitazone.

The transactivation effects of **8** on PPAR-α, PPAR-β/δ, and PPAR-γ were further verified by using SH-SY5Y neuroblastoma cells. Although less potent compared to the positive controls, **8** activated PPAR-γ in a dose-dependent manner (Figure 3A,C). Again, no PPAR-β/δ activity was observed (Figure 3B). Accordingly, compound **8** was expected to be a partial PPAR-γ agonist (with less PPAR-α activation) in SH-SY5Y neuroblastoma cells.

### 2.3. Docking Analysis of Compound ***8*** with PPAR-α, -β/δ, and -γ

**Figure 4:**
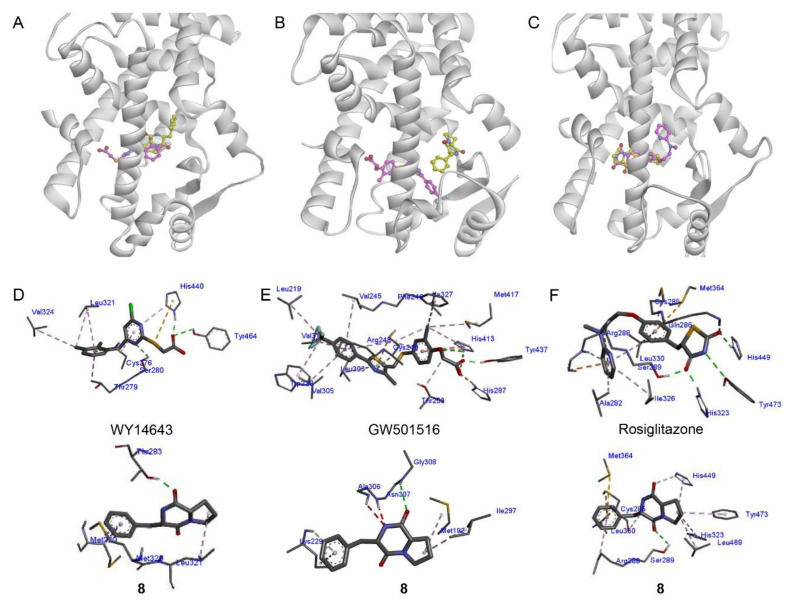
Docking analysis of compound **8** with PPAR-α (**A**), -β/δ (**B**), and -γ. (**C**). Compound **8** docked into PPAR-α ((PDB: 4BCR), -β/δ (5U46), and -γ (2PRG). The structure of **8** is shown in yellow, whereas the standard agonists WY14643, GW501516, and rosiglitazone are shown in pink. (**D**–**F**) The binding interactions of **8** with PPAR-α, -β/δ, and -γ in comparison with WY14643, GW501516, and rosiglitazone, respectively. H-bonds and hydrophobic interactions are shown in red and pink, respectively. PPAR, peroxisome proliferator-activated receptor.

Docking simulations between **8** and PPAR-α, PPAR-β/δ, and PPAR-γ were performed to assess the possible binding pose and receptor affinity. Compound **8** and PPAR-α agonist WY14643 occupied the same ligand-binding domain of PPAR-α (PDB code: 4BCR) (Figure 4A), with binding affinities of −8.3 and −7.7 kcal/mol, respectively (Table S1). Compound **8** showed partial overlap with WY14643 in the binding pocket of PPAR-α. WY14643 formed hydrogen bonds with Tyr^464^, His^440^, and Ser^280^ and demonstrated hydrophobic interactions with Val^324^, Leu^321^, Cys^276^, and Thr^279^. However, **8** formed a hydrogen bond only with Thr^283^ and demonstrated hydrophobic interactions with Leu^321^, Met^320^, and Met^220^ (Figure 4D). As for PPAR-β/δ (PDB code: 5U46), **8** was predicted to bind to a different site from that of GW501516 (Figure 4B) and no identical amino acid residue interaction was determined between **8** and GW501516 (Figure 4E and Table S2). This result was in line with the moderate activity of **8** against PPAR-β/δ. Similar to rosiglitazone, **8** was predicted to bind to the same ligand-binding domain of PPAR-γ (PDB code: 2PRG) (Figure 4C). Rosiglitazone forms hydrogen bonds with key amino acids Tyr^473^, His^323^, His^449^, Ser^289^, Gln^286^, and Arg^288^, with a binding affinity of −8.6 kcal/mol (Figure 4F and Table S3). However, **8** was speculated to form hydrogen bonds with His^449^, Ser^289^, and Cys^285^ residues, with a binding affinity of −7.5 kcal/mol (Figure 4F). As a PPAR-γ full agonist, rosiglitazone interacts with three key amino acids Tyr^473^, His^323^, and His^449^ to stabilize the AF-2 surface via H-bonding [50]; in particular, H-bonding with Tyr^473^ promotes the active conformation of helix 12 (H12) and activates PPAR-γ [51]. Compound **8** was expected to be a partial PPAR-γ agonist given the lack of interaction with Tyr^473^ [52].

### 2.4. Protective Effects of Compound ***8*** against H_2_O_2_-Induced Cell Injury

Activation of PPAR reportedly demonstrates beneficial effects in neurodegenerative diseases and CNS injury [53]; accordingly, ligands targeting PPARs are considered potential therapeutics in these pathologies. Thyrotropin-releasing hormone (TRH), which is a neural tripeptide amide, was first characterized in the hypothalamus and afforded neuroprotective effects in CNS trauma [54,55]. In addition to its neuroprotective effect, TRH exhibits physiological effects that may be undesirable for the treatment of neurotrauma [56]. Metabolic products of TRH, such as cyclo-(His-Pro) (CHP), protect cells against H_2_O_2_-induced injury by inhibiting oxidative stress [57]. The synthetic CHP mimetics, cyclo-[(*R*)-3′,5′-di-tert-butyl-Tyr-L-Pro] and cyclo-[(*S*)-3′,5′-di-tert-butyl-Tyr-L-Pro], reportedly inhibited neuronal cell death in a traumatic injury model [58]. Based on the common structural features of these neuroprotective compounds, a pharmacophore model was generated to assess neuroprotective effects of DKPs [59] (Figure S2A). Compound **8** was found to possess common neuroprotective structural features and was mapped onto the active pharmacophore model (Figure S2B). Therefore, compound **8** may be worth investigating for its potential neuroprotective effects.

**Figure 5:**
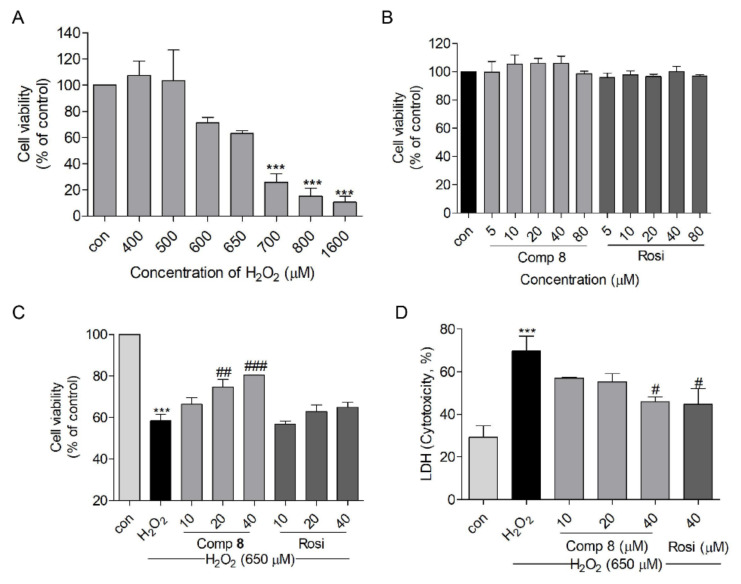
The neuroprotective effect of compound **8** against H_2_O_2_-induced damage in SH-SY5Y cells. (**A**) H_2_O_2_- induced cytotoxicity in SH-SY5Y cells at 15 h. (**B**) The viability of SH-SY5Y cells when treated with **8** or rosiglitazone for 10 h. (**C**) The protective effects of **8** and rosiglitazone against H_2_O_2_-induced damage in SH-SY5Y cells. (**D**) LDH release with **8** or rosiglitazone treatment in H_2_O_2_-treated SH-SY5Y cells. *** *p* < 0.001 compared with control. ^#^ *p* < 0.05, ^##^ *p* < 0.01, ^###^ *p* < 0.001 vs. H_2_O_2_. LDH, lactate dehydrogenase; Rosi, rosiglitazone.

The neuroprotective effect of **8** was evaluated using an H_2_O_2_-induced SH-SY5Y cell injury model, compared with the positive control rosiglitazone [21,60]. Before performing the neuroprotection assay, SH-SY5Y cells were exposed to various concentrations of H_2_O_2_, **8**, and rosiglitazone to determine appropriate concentrations for the assay. Concentrations approximating the IC_50_ values of H_2_O_2_ (650 μM) and the non-cytotoxic concentrations (10, 20, and 40 μM) of **8** and rosiglitazone were selected to perform the neuroprotection assay (Figure 5A,B). Pretreatment with **8** induced a dose-dependent increase in cell viability up to 66.4%, 74.6%, and 80.4% (at 10, 20, and 40 μM, respectively), revealing a potency higher than that of rosiglitazone (Figure 5C). In addition, a lactate dehydrogenase (LDH) release assay was performed to demonstrate this protective effect. The pretreatment with **8** or rosiglitazone decreased the H_2_O_2_-induced cytotoxicity to 45.9% and 44.8%, respectively, at a concentration of 40 μM (Figure 5D).

The blood–brain barrier (BBB) is a highly selective semipermeable barrier comprising endothelial cells, which prevents solutes in circulating blood from non-selectively crossing into the CNS where neurons reside [61]. As a possible neuroprotective agent, the BBB permeability of **8** was predicted using PreADMET [62] and the brain to the blood concentration ratio of **8** was determined as 0.558621 (Figure S3A); this was higher than that of CHP (0.140492) (Figure S3B). CHP reportedly accumulates in the CNS regardless of its low entry rate owing to its long half-life and marked resistance to enzymatic degradation [63]. Therefore, compound **8** could maintain a precise CNS concentration owing to its high stability against enzymatic hydrolysis [32].

### 2.5. Effects of Compound ***8*** on H_2_O_2_-Induced Apoptosis

**Figure 6:**
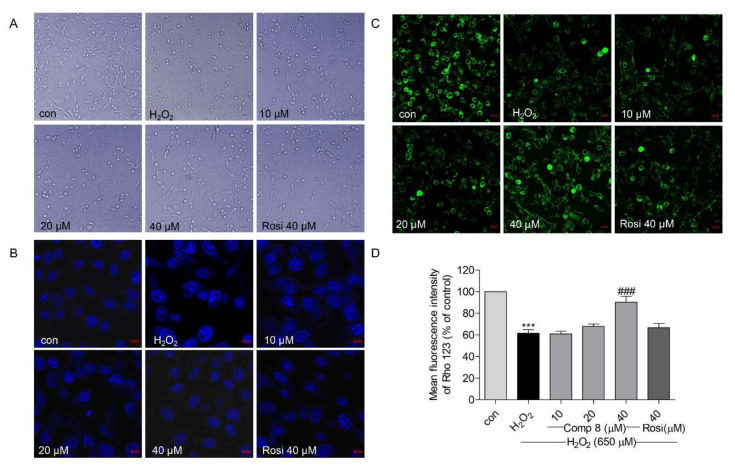
(**A**) The effects of compound **8** on H_2_O_2_-induced morphological changes in SH-SY5Y cells. (**B**) The effect of **8** on H_2_O_2_-induced nuclear condensation using the Hoechst 33342 staining assay. SH-SY5Y cells were pretreated with **8** for 10 h and exposed to H_2_O_2_ for an additional 15 h. (magnification, ×40). (**C**) The effect of **8** on the mitochondrial membrane potential (MMP) in SH-SY5Y cells. Rhodamine 123 was used to analyze the MMP. Loss of MMP results in mitochondrial damage and cell death. The green fluorescence shows live cells. Rhodamine 123 accumulates in the mitochondria of live cells. (magnification, ×40). (**D**) Quantitative presentation of the MMP change in SH-SY5Y cells. *** *p* < 0.001 compared with control. ^###^ *p* < 0.001 vs. H_2_O_2_. Rosi, rosiglitazone.

It is well-known that H_2_O_2_ is an effective mediator of oxidative stress and cell apoptosis, especially in the mitochondria of SH-SY5Y cells [64,65]. Herein, we microscopically examined morphological changes (H_2_O_2_-induced apoptosis) in SH-SY5Y cells, which appeared shrunken and round with apoptotic bodies when treated with 650 μM H_2_O_2_ (Figure 6A); however, pretreatment with **8** (10, 20, and 40 μM) and rosiglitazone (40 μM) suppressed these H_2_O_2_-induced changes (Figure 6A). Furthermore, pretreatment with **8** (10, 20, and 40 μM) and rosiglitazone (40 μM) suppressed H_2_O_2_-induced chromatin condensation (Figure 6B).

Mitochondrial membrane potential (MMP, ΔΨm) can regulate matrix configuration and cytochrome C release and MMP levels are reduced during apoptosis [66]. MMP loss is considered to induce cell death by damaging the mitochondria [67,68]. Rhodamine 123 (Rho 123) is employed as a probe to monitor MMP; the Rho 123 fluorescence decay rate corresponds to the MMP [69]. As shown in Figure 6C,D, the Rho 123 fluorescence intensity was significantly reduced in the H_2_O_2_-treated group; however, pretreatment with **8** (10, 20, and 40 μM) and rosiglitazone (40 μM) inhibited MMP loss. In addition, the effect of **8** was more potent than that of rosiglitazone at the same treatment concentration (40 μM). These results indicated that compound **8** suppressed H_2_O_2_-induced apoptosis in SH-SY5Y cells.

### 2.6. Effects of Compound ***8*** on H_2_O_2_-Induced Oxidative Stress

**Figure 7:**
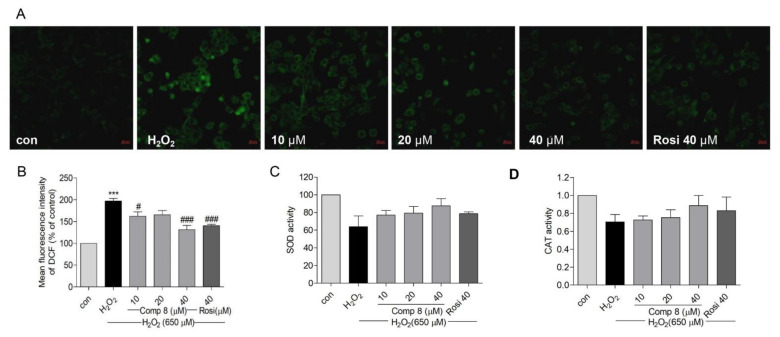
The effects of compound **8** on ROS generation and SOD and CAT activities in SH-SY5Y cells. (**A**) Suppression of ROS generation. SH-SY5Y cells were seeded in confocal dishes and pretreated with **8** or rosiglitazone for 10 h, followed by treatment with H_2_O_2_ for 14 h. ROS was visualized as green fluorescence using the DCFDA staining. (magnification, ×40). (**B**) Quantitative presentation of the ROS in SH-SY5Y cells. (**B**–**D**) shows SOD and CAT activities after treatment with **8**. *** *p* < 0.001 compared with control. ^#^ *p* < 0.05, ^###^ *p* < 0.001 vs. H_2_O_2_. ROS, reactive oxygen species; SOD, superoxide dismutase; CAT, catalase; Rosi, rosiglitazone.

Oxidative stress generates reactive oxygen species (ROS) and disrupts mitochondrial membrane permeability and mitochondrial defense systems; theses are known features that underlie the development of neurodegenerative diseases [70]. Apart from the generation of endogenous ROS, the mitochondria also act as a ROS target via feedback [71]. Oxidative stress directly targets mitochondria to induce apoptotic cell death [71]. As shown in Figure 7A,B, pretreatment with **8** (10, 20, and 40 μM) or rosiglitazone (40 μM) decreased H_2_O_2_-induced [72] ROS generation in SH-SY5Y cells.

Superoxide dismutase (SOD) is a metalloenzyme that plays a vital role against oxidative stress in the body [73]. SOD scavenges ROS to attenuate cell death [72]. Catalase (CAT) is the second most abundant enzymatic antioxidant that decomposes ROS [74,75]. Both SOD and CAT are the first lines of defense against free radical-induced tissue damage [76]. The treatment of SH-SY5Y cells with **8** (10, 20, and 40 μM) or rosiglitazone (40 μM) increased SOD and CAT enzyme levels but the activity was not significant (Figure 7C,D). These results suggested that compound **8** could suppress H_2_O_2_-induced oxidative stress by attenuating ROS generation in SH-SY5Y cells.

### 2.7. Effects of Compound ***8*** on H_2_O_2_-Induced Apoptosis-Related Proteins

**Figure 8:**
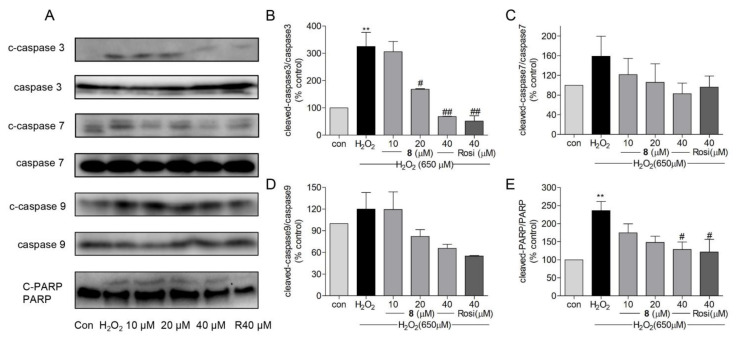
(**A**) The effects of compound **8** on protein levels of mitochondria-related apoptotic proteins, such as cleaved-caspase 3, 7, 9, and cleaved-PARP. (**B**–**E**) The quantitative analysis of cleaved-caspase 3, 7, 9, and cleaved-PARP compared with their inactive zymogens. ** *p* < 0.01 compared with control. ^#^ *p* < 0.05, ^##^ *p* < 0.01 vs. H_2_O_2_. PARP, poly (ADP-ribose) polymerase; Rosi, rosiglitazone.

Under oxidative stress, ROS-induced cell death is reportedly associated with caspase-activated apoptosis [72]. Activation of caspases is related to mitochondria-dependent apoptosis [71]. Morphological changes in mitochondria and ROS generation are mediated via caspase 9 [77]. Caspase 9 activates caspase 3, which is essential for brain development and contributes to apoptosis [77,78]. In addition, caspase 3 is responsible for the cleavage and activation of poly (ADP-ribose) polymerase (PARP), which activates DNA strand breakage [79]. In addition to caspase 3, caspase 9 also activates caspase 7 [77,80], which is a pivotal mediator of MMP loss (ΔΨm). Procaspases are inactive zymogens that need to be activated through cleavage [81]. We measured protein levels of cleaved-caspase 3, 7, and 9 and cleaved-PARP. As shown in Figure 8A–E, the ratio of cleaved-caspase 3 and cleaved-PARP to their inactive zymogens was significantly decreased when treated with **8** or rosiglitazone. Even though the error range of the data of caspases 7 and 9 (Figure 8C,D) was large, the trend of caspase inhibition by compound **8** can be observed. These results indicated that compound **8** could reduce the protein levels of cleaved-caspase 3 and cleaved-PARP in H_2_O_2_-induced damage in SH-SY5Y cells.

### 2.8. Effects of Compound ***8*** on NF-κB Activation and Nuclear Translocation

**Figure 9:**
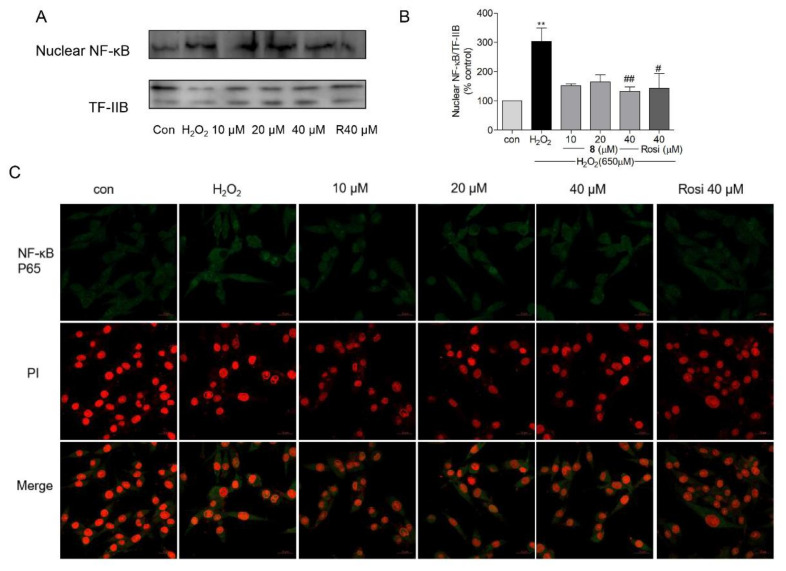
The effects of compound 8 on NF-κB activation. (**A**) The endonuclear level of NF-κB. (**B**) Western blotting was performed to analyze the level of NF-κB in the nucleus. (**C**) Confocal image of endonuclear NF-κB. The activation and translocation of NF-κB were analyzed using the immunofluorescence staining assay (magnification, ×40). The scale bar represents 20 µm. NF-κB, nuclear factor-kappa B; Rosi, rosiglitazone. ** *p* < 0.01 compared with control. ^#^ *p* < 0.05, ^##^ *p* < 0.01 vs. H_2_O_2_.

In patients with PD, elevated nuclear translocation of NF-κB has been observed in dopaminergic neurons [82]. In Aβ_25–35_-exposed rats (experimental AD model), IκB-α degradation was found to be enhanced; however, the neuroprotective agent sodium hydrosulfide, which enhances protein levels of PPAR-α and PPAR-γ, can block IκB-α degradation (i.e., NF-κB activation) [83]. NF-κB, which is a crucial mediator of host defense against pathogens, is activated by various stimuli, such as inflammatory factors or oxidants [84]. After activation of latent NF-κB in the cytoplasm, the NF-κB complex is translocated into the nucleus, thereby promoting NF-κB-regulated gene expression [84,85]. PPAR-γ can block tissue injury by suppressing the NF-κB pathway to decrease inflammation while promoting the nuclear factor erythroid 2-related factor 2 (Nrf2)/antioxidant response element (ARE) axis to reduce oxidative stress [86]. As an oxidant, H_2_O_2_ can promote NF-κB p65 activation and nuclear translocation in SH-SY5Y cells [87]. In the present study, western blot and immunofluorescence assays were performed to measure the activation and endonuclear translocation of NF-κB p65 after treatment with **8**. As shown in Figure 9C, the immunofluorescent staining assay revealed that **8** suppressed H_2_O_2_-induced NF-κB activation and endonuclear translocation. For further confirmation, the Western blot assay was performed and H_2_O_2_ induced the nuclear translocation of NF-κB p65, but **8** (10, 20, and 40 μM) and rosiglitazone (40 μM) decreased the nuclear protein level of NF-κB (Figure 9A,B). These results suggested that compound **8** inhibited NF-κB activation and its translocation through the activation of PPAR-γ.

## 3. Materials and Methods

### 3.1. Materials

WY14643, GW501516, rosiglitazone, MTT, H_2_O_2_, cytotoxicity detection kit PLUS (LDH), Hoechst 33342, and 2,7-dichlorofluorescein diacetate (DCFDA) were purchased from Sigma-Aldrich (St. Louis, MO, USA). Rhodamine 123 was purchased from Enzo Life Sciences, Inc. (Burlington, ON, Canada). The SOD and CAT assay kits were purchased from DoGenBio (Guro-gu, Seoul, Korea). Monoclonal rabbit antibodies for cleaved-caspase 3, 7, and 9; cleaved-PARP; caspase 3 and 7; and PARP, as well as monoclonal mouse antibodies for caspase 9 were purchased from Cell Signaling Technology (Beverly, MA, USA) and the dilution was 1:1000. High performance liquid chromatography (HPLC) was performed using a Gilson 307 pump, Shodex RI-71 detector, and ODS column (YMC-Triart C18, 250 × 10.0 mm, i.d. 5 μm). ^13^C NMR spectra were obtained using a Varian UNITY 400 spectrometer and ^1^H NMR spectra were recorded using a Varian INOVA 500 spectrometer. Optical rotation was detected using a Jasco P-1020 polarimeter.

### 3.2. Isolation of 2,5-DKPs

The ethyl acetate extract of the jellyfish-derived fungus *A. flavus* was subjected to ODS column and HPLC separation to obtain eight 2,5-DKPs (**1–8**) (Figure 1). It was not requisite to acquire the approval of ethical commission for the isolation of the fungus from the jellyfish *Aurelia aurita*.

Cyclo-(L-Pro-L-Pro) (1): 0.6 mg. [α]${}_{D}^{26}$ = −114.7 (1 mg/mL, MeOH). ^1^H NMR (500 MHz, CD_3_OD): δ 4.36 (t, J = 7.9 Hz, 2H), δ 3.50–3.54 (m, 4H), δ 2.27–2.34 (m, 2H), and δ 1.94–2.13 (m, 6H) ppm. ^13^C NMR (400 MHz, CD_3_OD): δ 168.49, 61.73, 46.19, 28.7, and 24.15 [45].

Cyclo-(L-Pro-L-Tyr) (2): 1.84 mg. [α]${}_{D}^{26}$ = −58.3 (1 mg/mL, MeOH). ^1^H NMR (500 MHz, CD_3_OD): δ 7.05 (d, J = 8.2 Hz, 2H), δ 6.72 (d, J = 8.2 Hz, 2H), δ 4.38 (t, J = 7.4 Hz, 1H), δ 4.03–4.10 (m, 1H), δ 3.49–3.65 (m, 2H), δ 3.07 (qd, J = 14.3, 4.7 Hz, 2H), δ 2.09–2.14 (m, 1H), δ 1.78–1.86 (m, 2H), and δ 1.20–1.28 (m, 1H) ppm. ^13^C NMR (400 MHz, CD_3_OD): δ 170.80, 166.98, 157.71, 132.07, 127.65, 116.24, 60.07, 57.91, 45.93, 37.66, 29.39, and 22.74 [46].

Cyclo-(L-Pro-L-Val) (3): 3.96 mg. [α]${}_{D}^{26}$ = −115.6 (1 mg/mL, MeOH). ^1^H NMR (500 MHz, CD_3_OD): δ 4.22 (t, J = 7.4 Hz, 1H), δ 4.04–4.07 (m, 1H), δ 3.50–3.61 (m, 2H), δ 2.51 (dt, J= 12.0, 6.6 Hz, 1H), δ 2.31–2.39 (m, 1H), δ 2.00–2.08 (m, 1H), δ 1.90–2.00 (m, 2H), δ 1.11 (d, J = 6.8 Hz, 3H), and δ 0.95 (d, J = 6.8 Hz, 3H) ppm. ^13^C NMR (400 MHz, CD_3_OD): δ 171.31, 166.32, 60.32, 58.80, 44.94, 28.71, 28.29, 22.01, 17.60, and 15.44 [45].

Cyclo-(L-4-OH-Pro-L-Leu) (4): 3.58 mg. [α]${}_{D}^{26}$ = −125.0 (1 mg/mL, MeOH). ^1^H NMR (500 MHz, CD_3_OD): δ 4.51–4.56 (m, 1H), δ 4.46–4.50 (m, 1H), δ 4.16–4.21 (m, 1H), δ 3.68 (dd, J = 12.7, 3.7 Hz, 1H), δ 3.45 (d, J = 12.7 Hz, 1H), δ 2.30 (dd, J = 13.0, 6.2 Hz, 1H), δ 2.04–2.16 (m, 1H), δ 1.85–1.98 (m, 2H), δ 1.46–1.58 (m, 1H), and δ 0.94–1.02 (m, 6H) ppm. ^13^C NMR (400 MHz, CD_3_OD): δ 173.02, 169.01, 69.11, 58.71, 55.17, 54.64, 39.43, 38.18, 25.79, 23.27, and 22.20 [45].

Cyclo-(L-Leu-L-Tyr) (5): 1.06 mg. [α]${}_{D}^{26}$ = +69.4 [1 mg/mL, MeOH]). ^1^H NMR (500 MHz, CD_3_OD): δ 7.01 (d, J = 8.4 Hz, 2H), δ 6.72 (d, J = 8.4 Hz, 2H), δ 4.25 (t, J = 3.7 Hz, 1H), δ 3.67 (dd, J = 10.0, 4.3 Hz, 2H), δ 3.22 (dd, J = 13.9, 3.5 Hz, 1H), δ 2.84 (dd, J = 13.9, 3.5 Hz, 1H), δ 1.39–1.50 (m, 1H) δ 0.90 (ddd, J = 13.9, 9.7, 4.3 Hz, 1H), δ 0.76 (dd, J = 9.7, 6.6 Hz, 6H), and δ 0.14 (ddd, J = 13.9, 9.7, 4.3 Hz, 1H) ppm. ^13^C NMR (400 MHz, CD_3_OD): δ 169.10, 165.79, 158.09, 132.74, 127.06, 116.43, 57.65, 54.18, 45.26, 39.45, 24.72, 23.36, and 21.41 [47].

Cyclo-(L-Tyr-L-Val) (6): 0.79 mg. [α]${}_{D}^{26}$ = +230.3 (1 mg/mL, MeOH). ^1^H NMR (500 MHz, CD_3_OD): δ 7.04 (d, J = 8.3 Hz, 2H), δ 6.72 (d, J = 8.3 Hz, 2H), δ 4.25 (t, J = 4.5 Hz, 1H), δ 3.63–3.66 (m, 1H), δ 3.15 (dd, J = 14.0, 5.3 Hz, 1H), δ 2.95 (dd, J = 14.0, 4.4 Hz, 1H), δ 1.67 (m, 1H), δ 0.84 (d, J = 7.0 Hz, 3H), and δ 0.51 (d, J = 7.0 Hz, 3H) ppm. ^13^C NMR (400 MHz, CD_3_OD): δ 169.59, 166.94, 157.88, 132.41, 127.55, 116.39, 61.31, 57.52, 39.41, 33.38, 19.17, and 17.23 [48].

Cyclo-(L-4-OH-Pro-L-Phe) (7): 9.95 mg. [α]${}_{D}^{26}$ = −77.6 (1 mg/mL, MeOH). ^1^H NMR (500 MHz, CD_3_OD): δ 7.21–7.33 (m, 5H), δ 4.50 (td, J = 5.2 1.8 Hz, 1H), δ 4.39 (ddd, J = 11.9, 6.0, 1.9 Hz, 1H), δ 4.30 (t, J = 4.9 Hz, 1H), δ 3.73 (dd, J = 13.1, 5.1 Hz, 1H), δ 3.32–3.37 (m, 1H), δ 3.14–3.23 (m, 2H), δ 2.09 (dd, J = 13.0, 5.9 Hz, 1H), and δ 1.40 (td, J = 12.4, 4.6 Hz, 1H) ppm. ^13^C NMR (400 MHz, CD_3_OD): δ 171.04, 166.86, 137.22, 130.76, 129.26, 127.84, 68.33, 58.15, 57.40, 55.04, 38.65, and 37.79 [45].

Cyclo-(L-Pro-L-Phe) (8): 5.55 mg. [α]${}_{D}^{26}$ = −107.8 (1 mg/mL, MeOH). ^1^H NMR (500 MHz, CD_3_OD): δ 7.23–7.32 (m, 5H), δ 4.46 (t, J = 3.9 Hz, 1H), δ 4.09 (dd, J = 9.8, 7.2 Hz, 1H), δ 3.52–3.60 (m, 1H), δ 3.36–3.42 (m, 1H), δ 3.19 (d, J = 3.8 Hz, 2H), δ 2.08–2.15 (m, 1H), δ 1.79–1.86 (m, 2H), and δ 1.25 (p, J = 10.6 Hz, 1H) ppm. ^13^C NMR (400 MHz, CD_3_OD): δ 170.96, 166.93, 137.39, 131.04, 129.48, 128.09, 60.09, 57.69, 45.98, 38.19, 29.37, and 22.79 [45,49].

### 3.3. Molecular Docking Study

The crystal structures of PPAR-α, β/δ, and PPAR-γ with PDB codes 4BCR, 5U46, and 2PRG were downloaded from the Protein Data Bank [88]. Proteins were prepared using the Chimera 1.10.2 software package (National Institutes of Health, Bethesda, MD, USA) [89]. Ligand preparation and the addition of polar hydrogen, Kollman charges, setting grid box parameters for proteins, and docking calculations were performed using AutoDockTools 1.5.6 (The Scripps Research Institute, La Jolla, CA, USA) and AutoDock Vina 1.1.2 (The Scripps Research Institute) [90]. Discovery Studio 4.5 (NeoTrident Technology Ltd., Beijing, China) [91] was used to analyze the protein–ligand interactions.

### 3.4. Cell Culture and Cell Viability Assay

The SH-SY5Y and Ac2F cells were cultured in Dulbecco’s modified Eagle’s medium (Hyclone, Logan, UT, USA) supplemented with 10% fetal bovine serum (Gibco-BRL, NY, USA) and 1% penicillin/streptomycin and incubated at 37 °C with 5% CO_2_. Ac2F cells (1 × 10^4^ cells/well) and SH-SY5Y cells (1 × 10^4^ cells/well) were seeded into 96-well plates and incubated overnight. Ac2F cells were treated with **1–8** for 12 h in the free medium. SH-SY5Y cells were pretreated with **8** (10, 20, and 40 μM) and rosiglitazone (40 μM) for 10 h and subsequently treated with H_2_O_2_ (650 μM) for another 14 h. After treatment, 20 μL MTT (0.5 mg/mL) was added to each well and incubated for 3 h in the dark. Then, the supernatant was removed and 150 μL dimethyl sulfoxide was added to dissolve formazan crystals. A microplate reader (Elx 800, Bio-Tek, Winooski, VT, USA) at 490 nm was used to analyze the absorbance.

### 3.5. LDH Release

The SH-SY5Y cells were seeded into 96-well plates with the background control (contained the assay medium), low control (spontaneous LDH release), and high control (maximum LDH release) groups. The cells were pretreated with **8** at 10, 20, and 40 μM and rosiglitazone at 40 μM for 10 h and then treated with 650 μM H_2_O_2_ for another 14 h. The cytotoxicity detection kit^PLUS^ (LDH) (Sigma-Aldrich, St. Louis, MO, USA) was used to analyze H_2_O_2_-induced cytotoxicity and the protective effects of **8** and rosiglitazone, according to the manufacturer’s instructions. A microplate reader (Elx 800, Bio-Tek, Winooski, VT, USA) was used to analyze the absorbance.

### 3.6. Luciferase Assay

The SH-SY5Y and Ac2F cells were seeded in 48-well plates. When cell density reached 90% confluence, the plasmids pcDNA3, TK-PPRE, PPAR-α, β/δ, and PPAR-γ were transfected into cells using the free medium for 4 h (Ac2F) or 24 h (SH-SY5Y) (this experiment was performed as described in our previous report) [92]. After treatment, the free medium was removed and the cells were incubated with complete medium overnight. Transfected cells were treated with **1–8**, WY-14643, GW501516, or rosiglitazone for 6 h (Ac2F) or 24 h (SH-SY5Y). The cells were lysed and data values were measured using the ONE-Glo™ Luciferase Assay System regent with GloMax^®^-Multi Microplate Multimode Reader (Promega Co., Madison, WA, USA).

### 3.7. Hoechst 33342 Staining

The SH-SY5Y cells were seeded in confocal dishes and incubated overnight. The cells were pretreated with **8** and rosiglitazone for 10 h and then exposed to H_2_O_2_ for 14 h. After treatment, the cells were fixed with 10% formalin solution for 15 min and stained with Hoechst 33342 reagent (10 μg/mL) for 20 min in the dark. The cells were washed three times with phosphate-buffered saline (PBS) and then visualized using a ZEISS LSM 800 confocal microscope (Oberkochen, Baden-Württemberg, German).

### 3.8. MMP Assay

The SH-SY5Y cells were seeded in confocal dishes and treated with **8** (10, 20, and 40 μM) and rosiglitazone (40 μM) for 10 h and then exposed to H_2_O_2_ for another 14 h. Next, the cells were stained with Rho 123 (10 μg/mL) for 20 min in the dark and analyzed using a ZEISS LSM 800 confocal microscope (Oberkochen, Baden-Württemberg, German) at 529 nm. The mean fluorescence intensity was quantified using ImageJ (National Institutes of Health, Bethesda, MD).

### 3.9. ROS Generation

The SH-SY5Y cells were seeded in confocal dishes and treated as described above. After treatment, the cells were washed with PBS and stained with DCFDA (5 μM) in free medium for 30 min in the dark. Finally, the cells were washed with PBS thrice and then analyzed using a ZEISS LSM 800 confocal microscope (Oberkochen, Baden-Württemberg, German) at 525 nm.

### 3.10. SOD and CAT Activities

The SH-SY5Y cells were seeded into 6-well plates and pretreated with **8** at 10, 20, and 40 μM and rosiglitazone (40 μM) for 10 h, followed by treatment with 650 μM H_2_O_2_ for an additional 14 h. After treatment, the cells were collected and lysed in the lysis buffer for 30 min on ice. The SOD and CAT activities were measured according to the manufacturer’s instructions of EZ-Catalase assay kit (DoGenBio Co., Ltd., Seoul, Korea) and the Superoxide dismutase assay kit was used (DoGenBio Co., Ltd., Seoul, Korea). SOD inhibition activity was determined at 450 nm using a microplate reader (Elx 800, Bio-Tek, Winooski, VT, USA). CAT reacted with H_2_O_2_ to produce water and oxygen and the unconverted H_2_O_2_ reacted with OxiRed^TM^ to generate a product measured at 570 nm using a microplate reader (Elx 800, Bio-Tek, Winooski, VT, USA).

### 3.11. Western Blotting

The SH-SY5Y cells were seeded on cell culture dishes and pretreated with **8** at 10, 20, and 40 μM and rosiglitazone (40 μM) for 10 h, followed by treatment with 650 μM H_2_O_2_ for an additional 14 h. After treatment, cells were collected and washed with PBS. Cell lysis buffer was added to the cell pellet to lyse the cells for 30 min on ice. The lysed cells were centrifuged at 13,000 rpm for 15 min and the supernatant protein concentration was measured using a BCA protein assay kit (Thermo Fisher Scientific, Waltham, MA, USA). Proteins were loaded and separated using sodium dodecyl sulfate-polyacrylamide gel electrophoresis. Then, proteins were transferred to PVDF membranes and blocked with 5% non-fat milk for 1 h at room temperature (25 °C). Next, the membranes were incubated with primary antibodies (cleaved-caspase 3, 7, and 9; cleaved-PARP; caspase 3, 7, and 9; and PARP) overnight. The membranes were washed three times with TBST and then incubated with secondary antibodies for 1 h at room temperature (25 °C). Finally, the membranes were washed three times with TBST and visualized using an ECL kit using the ChemiDoc™ Touch Imaging System (Bio-Rad Laboratories, Hercules, CA, USA).

### 3.12. Immunofluorescence Assay

The SH-SY5Y cells were seeded on confocal dishes and pretreated with **8** at 10, 20, and 40 μM and rosiglitazone (40 μM) for 10 h, followed by treatment with 650 μM H_2_O_2_ for another an additional 14 h. Then, the cells were fixed with 10% formalin solution and treated with 0.3% Triton X-100 for 15 min. The fixed cells were blocked with 10% BSA for 30 min at room temperature and incubated with the primary antibody anti-NF-κB overnight. The cells were washed three times with PBS and incubated with Alexa 488 secondary antibodies for 30 min at room temperature. Finally, 10 μg/mL of propidium iodide and 10 μg/mL RNase were added to confocal dishes and cultured for 30 min individually. The fluorescence of the SH-SY5Y cells was analyzed using a ZEISS LSM 800 confocal microscope (Oberkochen, Baden-Württemberg, German).

### 3.13. Statistical Analysis

Data analyses were performed using GraphPad Prism 5 (San Diego, CA, USA). Data values are presented as means ± standard error of the mean. One-way analysis of variance and Tukey’s HSD-post hoc test were used to analyze significant differences. * *p* < 0.05, ** *p* < 0.01, and *** *p* < 0.001 were used to determine statistical significance.

## 4. Conclusions

In the course of our search for natural PPAR agonists, eight 2,5-DKPs (**1**–**8**) were isolated from the jellyfish-derived fungus A. *flavus*. Compound **8** was selected as a partial PPAR-γ agonist and evaluated for neuroprotective effect using SH-SY5Y neuroblastoma cells. Compound **8** showed inhibition of H_2_O_2_-induced cell injury and ROS generation in SH-SY5Y cells, together with inhibition of H_2_O_2_-induced apoptosis and the loss of mitochondrial membrane potential. The activation of the apoptosis-related proteins—caspase 3 and PARP—was inhibited by **8**. In addition, compound **8** inhibited H_2_O_2_-induced activation and endonuclear translocation of NF-κB, which is a key physiological marker in patients with PD and experimental AD models. Therefore, compound **8**, which is a partial PPAR-γ agonist, was proposed to exert neuroprotective effects by modulating the NF-κB pathway. According to the in vitro results, compound **8** may be utilized as a partial PPAR-γ agonist for in vivo study in neurodegenerative diseases models.

## Supplementary Materials

The following are available online at https://www.mdpi.com/article/10.3390/md19080417/s1, Figure S1: The fungus *Aspergillus flavus* was identified by ITS gene sequences (GenBank accession no.KR011759), Figure S2: (A) Pharmacophore model for neuroprotective DKPs [1]. (B) Mapping of the pharmacophore onto cyclo-(L-Pro-L-Phe), Figure S3: The predicted ADME and BBB values of cyclo-(L-Pro-L-Phe) (A) and CHP (B) using PreADMET website, Table S1: PPAR-α docking simulation result, Table S2: PPAR-β/δ docking simulation result, Table S3: PPAR-γ docking simulation result.
