# Supplementary material for: Neuroprotective Effect of Cyclo-(L-Pro-L-Phe) Isolated from the Jellyfish-Derived Fungus Aspergillus flavus
Source: Mar Drugs. 2021 Jul 26;19(8):417. doi: 10.3390/md19080417 (PMC8401322; doi:10.3390/md19080417)
Supplement: Supplementary file 1 [file marinedrugs-19-00417-s001.zip › marinedrugs-1304570 - supplementary .pdf]

marine drugs

Article

Neuroprotective effect of cyclo-(L-Pro-L-Phe) from the Jellyfish-derived fungus *Aspergillus flavus*

Dan-dan Li , Ying Wang , Eun La Kim and Jee H. Jung *

[1] College of Pharmacy, Pusan National University, Busan 46241, Korea; leedany1992@gmail.com (D.-d.L.); wangrunhe829@gmail.com (Y.W.); eunlakim@gmail.com (E.L.K.)
* Correspondence: jhjung@pusan.ac.kr

Citation: Li, D.-d.; Wang, Y.; Kim, E.L.; Jung, J.H. Neuroprotective effect of cyclo-(L-Pro-L-Phe) from the Jellyfish-derived fungus *Aspergillus flavus*. **2021**, *19*, 417. https://doi.org/10.3390/md19080417

Received: 2 July 2021
Accepted: 22 July 2021
Published: 26 July 2021

Publisher's Note: MDPI stays neutral with regard to jurisdictional claims in published maps and institutional affiliations.

Copyright: © 2021 by the authors. Submitted for possible open access publication under the terms and conditions of the Creative Commons Attribution (CC BY) license (http://creativecommons.org/licenses/by/4.0/).

Supplementary Materials and Methods

Extraction and isolation

The fungus *Aspergillus flavus*, identified by ITS gene sequences (GenBank accession no.KR011759), was isolated from Jellyfish *Aurelia aurita* and saved in Marine Natural Product Laboratory, Pusan National University. The *Aspergillus flavus* fungus was cultured in 33 L YM medium (dissolved in 75% seawater) which included yeast extract (3.0 g/L), malt extract (3.0 g/L), peptone (5.0 g/L), and dextrose (10.0 g/L) for 21 days in a shaker platform with 130 rpm. After culture, the crude extract was extracted by EtOAC from the culture medium and separated by 90% methanol and *n*-hexane. The extract was collected from 90% methanol layer and eluted with 20%-100% methanol in step-gradient medium-pressure liquid chromatography (MPLC) (ODS-A, 120 Å, S-30/50 mesh). After that, we got 28 fractions. Fraction 5 (266.8 mg) was separated by RP-HPLC (YMC-triart C18, 250 × 10.0 mm, i.d. 5 μm) in 20% methanol / water, and 7 pure compounds (com 1: 0.6 mg; com 2: 1.84 mg; com 3: 3.96 mg; com 4: 3.58 mg; com: 1.06 mg; com 6: 0.79 mg; com 7: 9.95 mg) were obtained. Com 8 (5.55 mg) was got from fraction 10 using RP-HPLC (YMC-triart C18, 250 × 10.0 mm, i.d. 5 μm) in 40% methanol / water.

Supplementary Results

Primer Information

Sequencing Primer Name Primer Sequences	PCR Primer Name Primer Sequences
ITS1 5' (TCC GTA GGT GAA CCT GCG G) 3'	ITS1 5' (TCC GTA GGT GAA CCT GCG G) 3'
ITS4 5' (TCC TCC GCT TAT TGA TAT GC) 3'	ITS4 5' (TCC TCC GCT TAT TGA TAT GC) 3'

Subject						Score		Identities	
Accession	Description	Length	Start	End	Coverage	Bit	E-Value	Match/Total	Pct.(%)
KY234273.1	Aspergillus flavus	819	4	815	99	1188	0.0	767/823	93

Kingdom	Family	Genus	Species
Eukaryota	-	Aspergillus	Aspergillus flavus

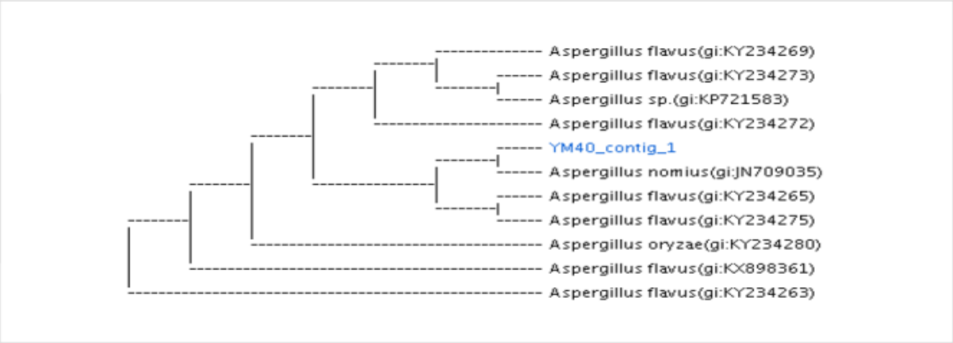

Figure 1. The fungus *Aspergillus flavus* was identified by ITS gene sequences (GenBank accession no.KR011759).

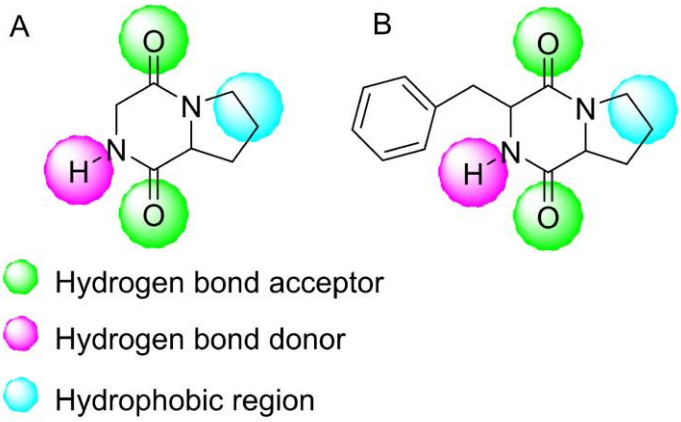

Figure 2. (**A**) Pharmacophore model for neuroprotective DKPs [1]. (**B**) Mapping of the pharmacophore onto cyclo-(L-Pro-L-Phe).

A **PreADMET website**

ID	Value
BBB	0.558621
Buffer_solubility_mg_L	12862.3
Caco2	21.8157
CYP_2C19_inhibition	Non
CYP_2C9_inhibition	Non
CYP_2D6_inhibition	Non
CYP_2D6_substrate	Non
CYP_3A4_inhibition	Non
CYP_3A4_substrate	Weakly
HIA	95.019141
MDCK	208.002
Pgp_inhibition	Non
Plasma_Protein_Binding	58.809343
Pure_water_solubility_mg_L	7309.04
Skin_Permeability	-3.95645
SKlogD_value	0.648710
SKlogP_value	0.648710
SKlogS_buffer	-1.278590
SKlogS_pure	-1.524050

BBB: in vivo blood-brain barrier penetration (C.brain/C.blood)= 0.558621

B

ID	Value
BBB	0.140492
Buffer_solubility_mg_L	86539.2
Caco2	15.0527
CYP_2C19_inhibition	Non
CYP_2C9_inhibition	Non
CYP_2D6_inhibition	Non
CYP_2D6_substrate	Non
CYP_3A4_inhibition	Non
CYP_3A4_substrate	Substrate
HIA	78.052390
MDCK	0.848824
Pgp_inhibition	Non
Plasma_Protein_Binding	15.841623
Pure_water_solubility_mg_L	347579
Skin_Permeability	-5.13313
SKlogD_value	-1.414550
SKlogP_value	-1.414550
SKlogS_buffer	-0.432480
SKlogS_pure	0.171360

BBB: in vivo blood-brain barrier penetration (C.brain/C.blood)= 0.140492

Figure 3. The predicted ADME and BBB values of cyclo-(L-Pro-L-Phe) (**A**) and CHP (**B**) using PreADMET website.

Table 1. PPAR-α docking simulation results.

compound	binding affinity (kcal/mol)	H-bond	Hydrophobic bond
WY14643	-8.3	Tyr^{464}, His^{440}, Ser^{280}	Val^{324}, Leu^{321}, Cys^{276}, Thr^{279}
**8**	-7.7	Thr283	Leu^{321}, Met^{320}, Met^{220}

Table 2. PPAR-β/δ docking simulation results.

compound	binding affinity (kcal/mol)	H-bond	Hydrophobic bond
GW501516	-10.5	Tyr^{437}, His^{413}, Thr^{253}	Leu^{219}, Trp^{228}, Val^{312}, Val^{305}, Val^{245}, Leu^{303}, Arg^{248}, Phe^{246}, Cys^{249}, Ile^{327}, Met^{417}, His^{413}
**8**	-8.4	Gly^{308}, Lys^{229}	Ile^{297}, Met^{192}, Lys^{229}

Table 3. PPAR-ϒ docking simulation results.

compound	binding affinity (kcal/mol)	H-bond	Hydrophobic bond
Rosig-litazoe	-8.6	Tyr^{473}, His^{323}, His^{449}, Ser^{289}, Gln^{286}, Arg^{288}	Arg^{288}, Ala^{292}, Ile^{326}, Leu^{330}, Cys^{285}

**8**	-7.5	His^{449}, Ser^{289}, Cys^{285}	Leu^{469}, Tyr^{473}, His^{323}, His^{449}, Arg^{288}, Leu^{330}

References:

1. Faden, A.I.; Movsesyan, V.A.; Fang, X.; Wang, S. Identification of novel neuroprotective agents using pharmacophore modeling. *Chem. Biodivers.* **2005**, *2*, 1564–1570.
